# Role of Radionuclide Therapy as Adjuvant to Palliative External Beam Radiotherapy for Painful Multiple Skeletal Metastasis

**DOI:** 10.4021/wjon235w

**Published:** 2010-08-29

**Authors:** Ayse Hicsonmez, Ozlem N. Kucuk, Meltem Nalca Andrieu, Yildiz Guney, Erkan Ibis

**Affiliations:** a Ankara University School of Medicine Department of Radiation Oncology, Turkey; b Ankara University School of Medicine Department of Nuclear Medicine, Turkey; c Ankara Oncology Hospital, Department of Radiation Oncology, Ankara, Turkey

**Keywords:** Bone metastases, Radiotherapy, Radionuclide therapy

## Abstract

**Background:**

The aim of this study was to evaluate the palliative efficacy of localized external radiotherapy (RT) combined with systemic radionuclide (RN) therapy in patients who had multiple painful osseous metastases of different primary origins.

**Methods:**

Thirty-three patients initially local external radiotherapy was delivered to the most symptomatic region in all patients. Then they received either Re 186 HEDP or Sm 153 EDTMP. The performance status was assessed according to ECOG scale. Before treatment, at the end of the radiotherapy and after the four weeks of systemic radionuclide therapy, analgesic intake and pain status were recorded by the RTOG scoring system, and EORTC QLQ C30 (Version 3.0 Turkish) questionnaire was performed to evaluate the quality of life.

**Results:**

Improved performances of 33.3% for post radiation therapy and 50% for post radionuclide therapy in the ECOG scale were observed. Statistically significant correlations were found between the primary origins and decreased pain and analgesic intake (p < 0.05), but no differences were observed on the self assessment quality of life questionnaire.

**Conclusions:**

Both Re 186 HEDP, Sm 153 EDTMP are effective and safe in bone pain palliation as an adjuvant to local field radiation therapy of breast and prostate cancer patients, who also continued to receive chemotherapy and/or hormontherapy.

## Introduction

Bone metastasis is a common, challenging problem in patients with advanced cancer. Although they are often clinically silent, they may lead to serious complications such as pain, fractures and hypercalcemia, which result in reduce performance status and decrease the quality of life. The life expectancy of the patients with bone metastases varies widely, depending on tumor types [1]. Management of bone pain can be maintained with analgesia, radiation, hormones, chemotherapy and surgery. Localized sites of bone involvement can be treated with surgery, radiofrequency ablation or external beam radiotherapy, whereas radiopharmaceuticals, hormones and chemotherapy are used to treat more diffuse bone involvement.

Radiation therapy and surgery are used for the treatment of localized bone metastases. The goal of localized irradiation is to relieve symptoms, restore function and prevent the sequelae of disease progression in the area treated [2-6]. Despite the lack of a dose response relationship for local field radiation therapy, different dose fractionations have been recommended [7]. Some patients with extensive disease are candidates for hemibody irradiation, however this treatment is associated with a high incidence of side effects [8]. Response rates are reported to be higher than 70%, and complete relief of pain has been achieved in 20% of patients [9, 10]. On the other hand, systemic radionuclide therapy represents a better approach for patients who have multiple bone metastatic sites. In recent years it has been employed with increasing frequency [11-14]. The advantages of targeted radionuclide therapy are the simultaneous treatment of all affected areas and the fact that it shows tumor specificity with relative sparing of the surrounding tissue. Patients can usually benefit from a single injection and pain relief may be obtained within the first week of treatment, which lasts for several months [15]. Compared with extended radiation therapy, radiopharmaceuticals can be given multiple times. They may be used in combination with other treatment methods and they have minimal adverse effects to healthy soft tissues adjacent to bone involvement [15]. The radioisotopes used in the treatment of metastatic bone disease are Phosphorus-32, Strontium-89, Samarium-153, Ethylene Diamine Tetramethylene Phosphoric acid (Sm 153 EDTMP), and Rhenium-186 Hydroxyethylidine Diphosphonate (Re 186 HEDP). The therapeutic effect of both Sm 153 EDTMP and Re 186HEDP is obtained by beta emission with a maximum of 1.07 MeV (maximal range 3 mm) for Re186 HEDP and beta max 0.8 MeV (maximal range 3.4 mm) for Sm153 EDTMP [13]. The evaluation of treatment induced pain relief remains problematic in clinical practice. There are in fact multiple reports which demonstrate differences between the doctor’s and the patient’s evaluation of the pain relief. For this reason, the self administrated questionnaire has been developed and is increasingly used in clinical trials [16].

The aim of this study was to evaluate the palliative efficacy of localized external radiotherapy (RT) combined with systemic radionuclide (RN) therapy in patients with multiple painful osseous metastases of different primary carcinoma.

## Patients and Methods

### Patients

Thirty-three patients who had multiple transient pains because of multiple skeletal metastases from different primary origins were eligible for this trial. The primary histology proved that malignancy was breast cancer in eight patients, prostate cancer in six, lung cancer in ten, gastro-intestinal system (GIS) cancer in three and various other types of cancer in six. A 99m Tc-methylenediphosphonate (MDP) bone scan was obtained in all patients before the radiotherapy and one week after systemic radionuclide therapy. The performance status was recorded according to the Eastern Cooperative Oncology Group (ECOG) scale. All patients were required to have a mandatory pain and narcotic evaluation using a scoring system validated through the Radiation Therapy and Oncology Group (RTOG) before treatment, at the end of radiotherapy and four weeks after radionuclide therapy. Pain and narcotic scores were obtained by multiplying severity and frequency. Pain severity was classified as: 0 (none), 1 (mild), 2 (moderate), or 3 (severe). Pain frequency was classified as: 0 (none), 1 (occasional = less than daily), 2 (intermittent = at least once daily), or 3 (constant). Drug severity was classified as 0: (not administered), 1 (analgesic), 2 (mild narcotic), or 3 (strong narcotic). Drug frequency was classified as: 0 (not administered), 1 (less than once per day), 2 (once per day), or 3 (twice or more per day). The patients’ characteristics are shown in table1.

### Treatment protocol

All patients received external local radiotherapy to a maximum of two sites. The local field size was chosen to include the most painful site and an appropriate margin. The patients were given three different doses; 30 Gy in 10 daily fractions, or 20 Gy in five fractions, or 8 Gy in one fraction. At the end of, or one week after, the radiotherapy, the patients received Re 186 HEDP = Rhenium 186 hidroxyethylydene disphosphonate (n = 11) or Sm 153 EDTMP = Samarium 153 ethylene diamine tetramethylene phosphate (n = 22). Each patient received 1,221 MBq Re 186 HEDP or 37 MBq/kg Sm153 EDTMP. To confirm RN treatment, bone scans were taken after 72 hours for Re 186 HEDP and 24 - 48 hours for Sm 153 EDTMP. All patients had received chemotherapy and/or hormontherapy and biphosphanate according to the therapeutic protocol.

The patients were issued with an EORTC QLQ-C30 (version 3.0 Turkish) questionnaire before the radiotherapy, at the end of the radiotherapy and four weeks after the radionuclide therapy.

As a second end point, overall survival (OS) and progression free survival (PFS) was reported. OS was calculated from the date of diagnosis of bone metastasis to the date of the last follow up. PFS was calculated from the date of radiation therapy to the date of physician-reported progression.

A response was defined as an improvement of the ECOG performance status by at least one level and symptomatic response reduction of the prescribed daily dose of analgesics and pain level compared with the pretreatment situation.

### Statistical analysis

All data was transferred to the SPSS version 10.1 for statistical analysis. Differences between pre-treatment and post-treatment ECOG, pain and analgesic scores were analyzed by Wilcoxon Signed Ranks. Survival and the duration of pain palliation were assessed by Kaplan-Meier curves. The differences in Kaplan Meier curves with respect to the primary tumors were evaluated by log rank tests. Differences of quality of life questionnaire variables were evaluated by *t*-tests. For statistical analysis P values less than 0.05 were considered significant.

## Results

### Patients

Fourteen patients with disseminated bone metastasis had breast (n = 8) or prostate cancer (n = 6), and 19 patients were affected from other cancers. Patients received the systemic radionuclide treatment on an outpatient basis whereas the radiation therapy was given on either an out- or in-patient basis (Table 1).

**Table 1:** The Patients’ Characteristics

Characteristic	n	%
Age	(35-82)	
Sex		
Female	20	60.6
Male	13	39.4
Primary		
Breast + Prostate	14	42.4
Others	19	57.6
ECOG		
≤ 2	10	30.3
> 2	23	69.7
Pain Score		
≤ 4	10	30.3
> 4	23	69.7
Analgesic score		
≤ 4	24	72.7
>4	11	33.3

### Clinical efficacy

In all patients the response rate was 33.3% at the end of RT and this increased to 50% four weeks after RN. The response rate in comparing the post radiation therapy to the initial evaluation after post RN was observed to be 50%. The response rates are shown in Table 2. Looking at the response rate with regard to site of primary tumor, the higher symptomatic improvement was obtained in patients with prostate and breast cancer than in patients with other cancer types. At the end of the RT, response to treatment was observed in 20% of the patients, four weeks after RN, therapy response was observed in 50% of the patients in breast and prostate cancer group versus 7.7% and 18.2% of the other patient groups, respectively (Table 3). Statistically significant correlations were found between the primary origins and decreased pain and analgesic intake (p < 0.05). Response rates were not related to previous chemotherapy and/or hormontherapy.

**Table 2:** Overall Symptomatic Response

	ECOG	Pain Score	Analgesic Score
Pre RT vs Post RT	0.003	0.000	0.002
Post RT vs Post RN	0.157	0.067	0.020
Pre RT vs Post RN	0.008	0.000	0.001

RT: radiotherapy; RN: radionuclide.

**Table 3:** Symptomatic Improvement Based on Primary Diagnosis

	Breast + Prostate	Other
Pre Treatment vs Post RT	20%	7.7%
Pre RT vs Post RN	57.1%	25%
Post RT vs Post RN	50%	18.2%

RT: radiotherapy; RN: radionuclide.

### Quality of life

Baseline quality of life questionnaires were assessable in 33 patients. At the end of RT and post RN, QLQ forms were available for 23 patients. However, no differences were observed on the self assessment quality of life questionnaire following the treatment. The differences in the global quality of life scale before RT after RN therapy and before and after RN therapy were found to be statistically significant (p = 0.001 and p = 0.003 respectively)

### Toxicity

Four patients (12%) experienced a flare reaction (defined by a transient increase in pain intensity after tracer administration). It occurred in the first week after the injection and disappeared spontaneously. No patient showed any appreciable change in vital signs or any clinically evident acute adverse effect. Bone marrow suppression was generally mild, reversible. There was no significant correlation between hematological toxicity and chemotherapy cycles and/or RT.

### Survival

**Figure 1:**
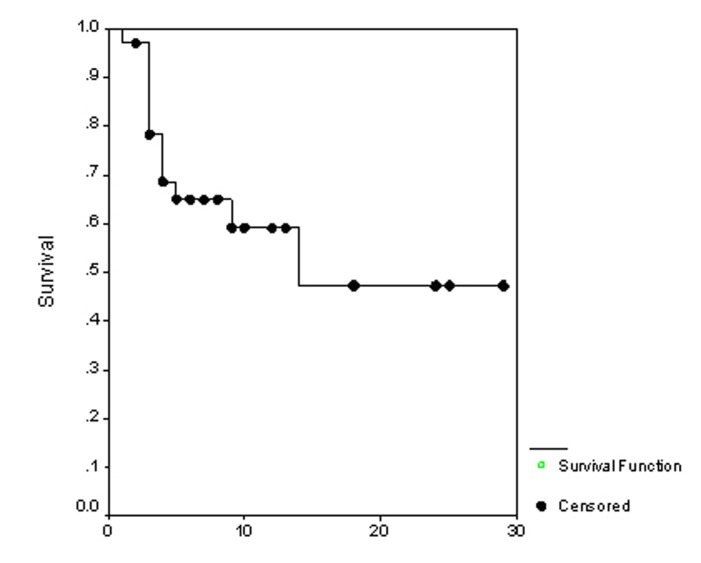
The overall survival.

**Figure 2:**
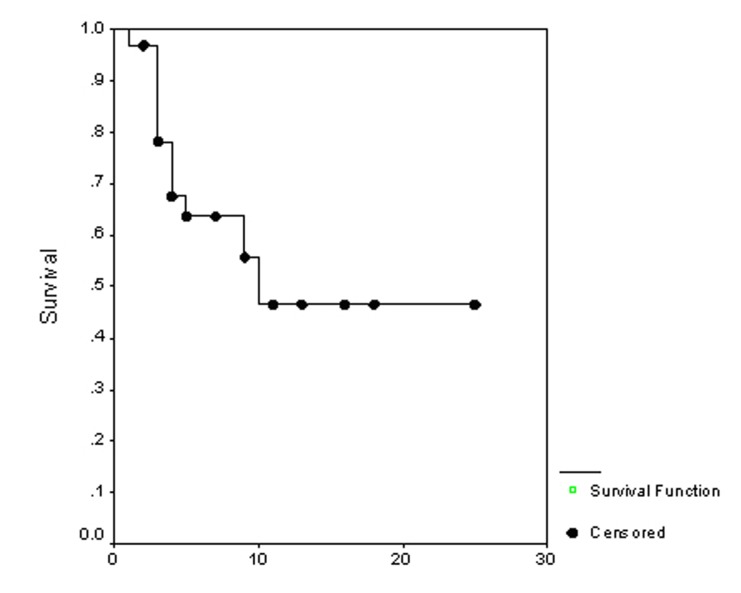
Progression free survival.

**Figure 3:**
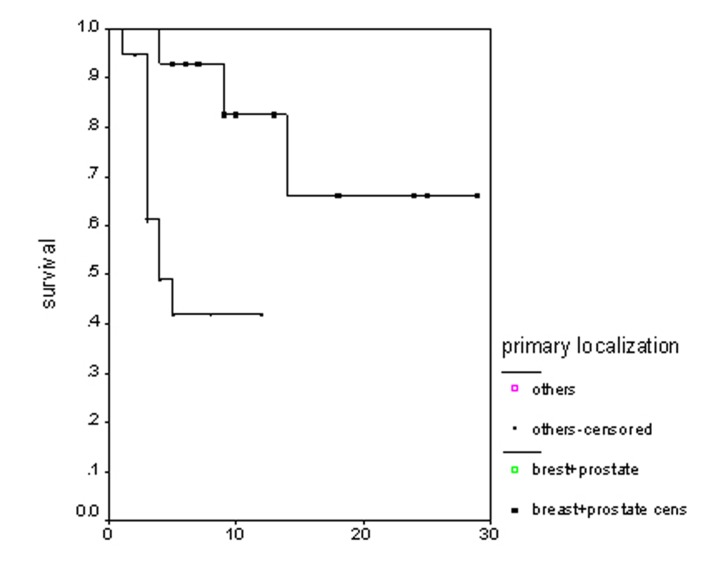
Overall survival irrespective of the treatment group.

**Figure 4:**
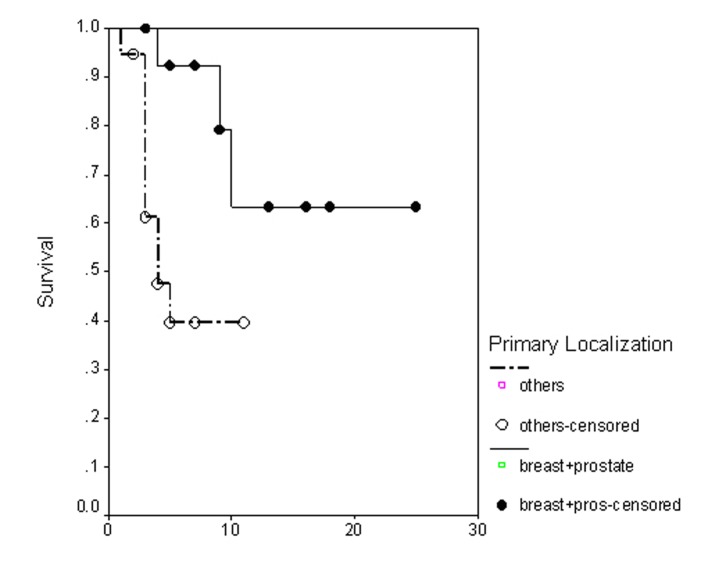
Progression free survival irrespective of the treatment group.

The overall survival (OS) for all patients was 17 months (Fig. 1). Progression free survival (PFS) for all patients was 10 months (Fig. 2). Irrespective of the treatment group, prostate and breast cancer patients had statistically significantly better overall survival and PFS than patients with other cancer types, with a respective OS of 22 and 6 months (p = 0.0062) (Fig. 3), PFS of 18 and 6 months (p = 0.0080) (Fig. 4).

## Discussion

Metastatic disease represents roughly 40-50% of oncology practice. Over 70% of patients with metastatic disease exhibit uncontrolled pain [17]. Pain management is often difficult in patients with bone metastasis. However, no optimal treatment strategy has been identified and studies which compare different treatment modalities are absent [18-20]. The cornerstone of malignant bone pain treatment has been radiation therapy. External radiotherapy options include local field as well as systemic approaches (half body radiation) in single or fractionated doses. Patients with bone metastasis due to breast and prostate cancer were believed to benefit most from radiation therapy and radionuclide therapy and were considered to be of main interest. But this study also provides a comparison with the other types of cancer. Radioisotopes can reduce pain and delay development of new painful sites [21]. Desirable characteristics of therapeutic radioisotopes include a high linear energy transfer; a shorter half life and the gamma emission. The gamma emission is appropriate for imaging with conventional scintigraphic cameras and enables the physician to confirm the delivery of the radiopharmaceutical to the targeted bone lesions. Presently, Sr 89, Re1 86 HEDP, Sm 153 EDTMP are the preferred radiopharmaceuticals [22, 23].

In our study, the overall clinical response with RT and with RN (Re 186 HEDP and Sm 153 EDTMP) were reported respectively as 33.3% and 50%. Patients with non breast and non prostate cancer might have negatively influenced our results. Our breast and prostate cancer patients showed better progression free survival and pain relief than the other cancer types. Theoretically, pain palliation in non prostate cancer could show peculiar features, as the pathologic microenviroments of bone metastases are quite different, with prostate cancer having a prevalently osteoblastic structure and the other cancers lytic or mixed patterns; especially breast cancer shows mixed features. The lytic pattern may result in a less suitable target for RN treatment. Liepe *et al* reported that both Re 186 HEDP and Sm 153 EDTMP, showed similar pain palliation effects in patients suffering from breast and prostate cancer [23]. In 21 studies, the overall clinical response rate with rhenium is reported to vary from 50% to 92%, in breast cancer patients with painful bone metastases [24]. Some investigators have found that response of Sr 89 to metastatic bone pain from prostate was better than in breast cancer [25].This may be due to the fact that osteoblastic metastases from prostate cancer respond better than osteolytic metastases from breast tumor [26]. Due to the mixed osteoblastic-osteolytic nature of the osseous metastases, the estimation of the pattern of the metastatic lesion is difficult. It has been reported that the application of a standard dose of Re 186 HEDP to patients with lung cancer and painful disseminated bone metastases had a significant pain alleviating effect. In estimating the osteoblastic and osteolytic element of the metastases, a careful selection of the patients is very important in order to achieve the optimum analgesic effect [27]. In this connection our patients underwent control bone scans before and after RN treatment. Extensive involvement with radiopharmaceuticals agents was observed. Meanwhile it was shown that RN treatment was effective.

Palliation of pain, or pain relief, is a very subjective response and has always been a difficult subject for research. The rate of response may also depend upon the pretreatment condition of the patient, the etiology of the bone metastases, the extent of the disease, and previous or concurrent local or systemic therapy given. Although there are standard ways of measuring pain using a pain scale, investigators often cannot calculate pain relief. The Visual Analogue Scale is probably the most accurate method to measure pain intensity and variations but, it is difficult to use in the elderly and in patients with poor compliance who represent the majority in the palliative care setting. Most studies have described only pain assessment, without adjustment for medication index and daily activities. Pain relief can be observed as a result of the increased use of analgesic. Any simultaneous change in the use of analgesics must be considered in the final response evaluation. Our experience with response evaluation is supported with changing pain, analgesic, and ECOG performance scales. Palmedo *et al* found a 60% response in breast cancer patients using a pain assessment through daily documentation of the visual analogue scale [28]. They also found that treatment with Re186 HEDP resulted in pain reduction if the patient experienced pain in a region where local external beam radiotherapy had previously been applied. Kucuk *et al* found an overall response rate of 67.5% with different types of cancer (prostate, breast, rectal, nasopharyngeal) using Re 186 HEDP [29]. The pain relief was assessed in accordance with the KPS index. Serafini reported that Sm153 EDTMP was efficient in relieving the pain of bone metastases in a variety of solid tumors [15]. This pain relief was accompanied by a significant decrease in opioid use. Re 186 HEDP, and Sm 153 EDTMP differ, especially in beta energy; Sm 153 EDTMP with a relative low maximum energy of 0.8 MeV and Re 186 HEDP with a higher maximum energy of 1.07 MeV. Some authors prefer the use of low beta energy emitters to reduce bone marrow toxicity in palliative treatment [30]. Liepe *et al* reported that data on the radiation absorbed dose of Re 188 HEDP showed comparable results to with other bone-seeking radionuclides with lower energies than 188Re-HEDP for 186Re- HEDP, 153Sm-EDTMP [31]. Our own data showed that Re 186HEDP and Sm 153EDTMP were well tolerated. There was no evidence of either local or systemic complications, and the flare was reversible in all patients who received palliative treatment. In this study, the global hematological toxicity of the treatment was low. No connection was observed between previous chemotherapy and treatment toxicity. Although most patients had previous chemotherapy, we did not observe any intolerable toxicity (defined as Grade 3). Since previous studies have shown that radionuclide therapy as an adjuvant to external beam radiotherapy or chemotherapy can both produce better palliative results and delay progression of disease, introduction of combination therapy protocols in routine practice is feasible and worthwhile [17]. Palmedo *et al* have reported similar results with breast cancer patients who had been treated with Re 186 HEDP [28]. However, another study has reported that the patients who had been treated with Sm 153 Lexidronam and RT or chemotherapy suffered Grade 3-4 leukocyte or platelet toxicities [15]. Yet studies using radiopharmaceuticals combined with chemotherapy have shown that pain response and survival were improved [12]. Patient selection criteria and pain relief evaluation were different but toxicity was significantly lower in our study. In other similar studies, Grade 2 or less hematologic toxicity has been reported for Sr 89 as adjuvant to palliative external beam radiotherapy [32].

To our knowledge, there are few studies about RN treatment as an adjuvant to external beam radiation for multiple painful osseous metastases in the literature. Hauswirth found a response rate of 59% and concluded that Re 186 HEDP can be used in conjunction with analgesic and external beam irradiation [33]. Porter & McEwan reported that a randomized controlled trial with the addition of Sr89 to external beam radiotherapy, involving 126 prostate patients, had no effect on survival, though the number of new sites of pain was significantly lower for the group receiving Sr89 [12]. In a Canadian study, Sr89 did not yield any additional effect at the external irradiated site but delayed and prevented new pain requiring RT at other sites [18]. In a randomized trial, Sr89 adjuvant to external radiotherapy did not seem to reduce the number of patients with subjective progression at three months [34]. In our study, the definition of progression included deterioration of the performance status, and pain increase with increased analgesic intake. Although the overall response rate was lower than in other studies, the progression free survival was better in our study. Local external radiotherapy was delivered to the most symptomatic region initially in all patients, who then received RN treatment. Because the time interval between RT and RN treatment was short, patient evaluation for initial response to RT, to RN or to both was difficult. After RN treatment, the patients were still under the palliative effect of RT. The reason for the long duration of palliation was the concomitant effect of RT and RN treatments. Life expectancy is an important criterion for treatment selection. A major problem is that pain relapse outside the irradiated area is likely to occur because the underlying disease is multifocal. The present study shows that breast and prostate cancer have longer OS and PFS, as expected. The development of new sites of pain can be delayed, and the requirement for additional RT and RN treatment reduced. Radionuclide therapy is the systemic use of radioisotopes for bone pain, and is an alternative for wide field irradiation which is known to have major disadvantages in terms of toxicity. However, individual studies are difficult to compare because various and different methods are used in the assessment of the clinical responses.

In conclusion, the results of this study demonstrate that both Re 186 HEDP and Sm 153 EDTMP are effective and safe in bone pain palliation as an adjuvant to local field radiation therapy of breast and prostate cancer. Re 186 HEDP and Sm 153 EDTMP show similar response rates, hematological toxicity is not clinically relevant, and chemotherapy is not impaired by RN treatment.
